# Brain Delivery of Cisplatin Using Microbubbles in Combination with Ultrasound as an Effective Therapy for Glioblastoma

**DOI:** 10.3390/ph16111599

**Published:** 2023-11-13

**Authors:** Fumiko Hagiwara, Daiki Omata, Lisa Munakata, Saori Kageyama, Kazuo Maruyama, Nobuki Kudo, Ryo Suzuki

**Affiliations:** 1 Laboratory of Drug and Gene Delivery Research, Faculty of Pharma-Science, Teikyo University, Tokyo 173-8605, Japan; 2 Laboratory of Pharmaceutics & Biopharmaceutics, Faculty of Pharma-Science, Showa Pharmaceutical University, Tokyo 194-8543, Japan; 3 Laboratory of Theranostics, Faculty of Pharma-Science, Teikyo University, Tokyo 173-8605, Japan; 4 Advanced Comprehensive Research Organization (ACRO), Teikyo University, Tokyo 173-0003, Japan; 5 Laboratory of Biomedical Engineering, Faculty of Information Science and Technology, Hokkaido University, Sapporo 060-0814, Japan

**Keywords:** blood–brain barrier, microbubble, ultrasound, drug delivery system, cisplatin, glioblastoma, theranostics

## Abstract

Glioblastoma is a highly invasive and fatal disease. Temozolomide, a blood–brain barrier (BBB)-penetrant therapeutic agent currently used for glioblastoma, does not exhibit sufficient therapeutic effect. Cisplatin (CDDP), a versatile anticancer drug, is not considered a therapeutic option for glioblastoma due to its low BBB permeability. We previously investigated the utility of microbubbles (MBs) in combination with ultrasound (US) in promoting BBB permeability and reported the efficacy of drug delivery to the brain using a minimally invasive approach. This study aimed to evaluate the feasibility of CDDP delivery to the brain using the combination of MBs and US for the treatment of glioblastoma. We used mice that were implanted with glioma-261 GFP-Luc cells expressing luciferase as the glioblastoma model. In this model, after tumor inoculation, the BBB opening was induced using MBs and US, and CDDP was simultaneously administered. We found that the CDDP concentrations were higher at the glioblastoma site where the US was applied, although CDDP normally cannot pass through the BBB. Furthermore, the survival was longer in mice treated with CDDP delivered via MBs and US than in those treated with CDDP alone or those that were left untreated. These results suggest that the combination of MBs and US is an effective antitumor drug delivery system based on BBB opening in glioblastoma therapy.

## 1. Introduction

Brain tumors can be classified into primary glioblastoma arising from the local tissue and metastatic glioblastoma originating from cancers at other sites. Gliomas are one of the most common primary tumors in the brain and present with symptoms such as persistent headache, nausea, vomiting, memory loss, and personality and visual changes [1,2,3]. Gliomas are categorized from grades 1 to 4 according to the World Health Organization classification of malignancies [4,5]. Glioblastoma, also known as grade 4 glioma, is the most malignant type, accounting for 36% of all gliomas [4]. Importantly, glioblastoma is highly fatal, with an average 5-year survival rate of less than 5% [6,7]. Chemotherapy is important for glioblastoma treatment due to its high invasiveness into the brain tissue, which is also associated with difficulty in its surgical removal [5,8].

The blood–brain barrier (BBB) restricts drug transport from peripheral blood to the brain tissue [9,10]. Temozolomide (TMZ), an oral alkylating agent that can cross the BBB, has nearly 100% bioavailability after oral administration in humans and is therefore used for chemotherapy in patients with glioblastoma [11]. However, glioblastoma sensitivity to TMZ is low, with response rates of 30–35% and insufficient therapeutic efficacy [5,6,12,13,14]. Cisplatin (CDDP) is a potent anticancer agent that is used for treating >50% of all cancer types [15]. The lower chloride ion concentration inside cells compared with the plasma has been reported to lead to the formation of CDDP aqua complexes within the cells [16]. After being taken up by vascular endothelial cells that form the BBB, CDDP forms aqua complexes and cannot pass through the cell membrane, limiting its migration into the brain tissue. The low BBB permeability of CDDP limits its applicability for patients with glioblastoma. Improving its BBB permeability could enhance the therapeutic efficacy of CDDP. Therefore, approaches that can improve BBB permeability for drug delivery, such as techniques that can open the BBB, are a focus of intense research [17].

Among the various methods to open the BBB is the osmotic approach, which utilizes the osmotic pressure difference caused by the administration of a hypertonic solution to open the BBB. However, this method can lead to cerebral edema as a side effect [18], which can occur as a result of glioblastoma as well [19,20,21,22]. Therefore, this approach may exacerbate cerebral edema when used to deliver anticancer drugs in patients with glioblastoma. Recently, attention has been paid to the combination of microbubbles (MBs) and ultrasound (US) due to its ability to open the BBB for drug delivery to the brain. MBs under the US field cause repeated expansion and compression and open the BBB by temporal disruption of tight junctions [23]. Additionally, focused US irradiation or the combination of MBs and US has been reported to promote transcytosis within endothelial cells [24]. This phenomenon could improve drug delivery to the brain. Therefore, the combined use of MBs and US to open the BBB may be a more suitable method for drug delivery to the brain for glioblastoma treatment than the osmotic method because it can reduce the risk of cerebral edema.

We have previously reported the development of MBs with an outer shell composed of lipids [25]. We also examined whether opening the BBB using MBs and US could be considered minimally invasive and efficient for drug delivery to the brain by investigating US irradiation conditions and inner gas types [26,27,28]. Therefore, we hypothesized that opening the BBB using MBs combined with US would allow CDDP to cross the BBB and reach the tumor tissue to exert its antitumor effect. In this study, we attempted to deliver CDDP to glioblastoma using the BBB-opening method with MBs and US. Furthermore, this study aimed to determine the utility of CDDP delivery for glioblastoma treatment.

## 2. Results and Discussion

### 2.1. Evaluation of the Sensitivity of Glioblastoma Cells to Anticancer Drugs

**Figure 1:**
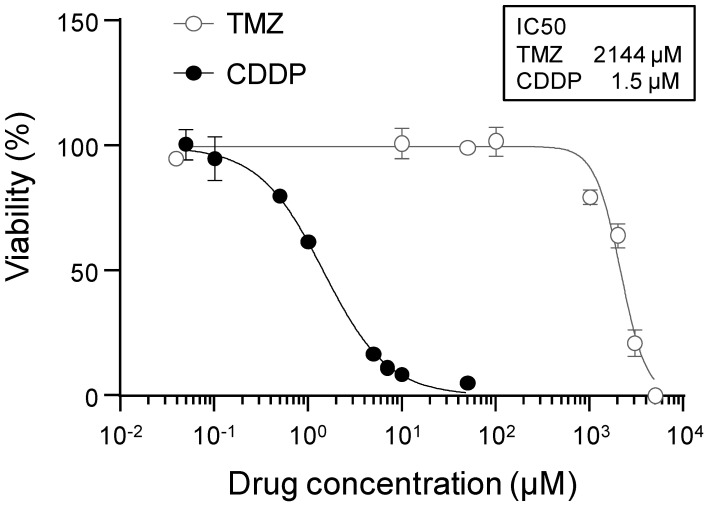
Sensitivity of glioblastoma cells to anticancer drugs. Glioma-261 GFP-Luc (3 × 10^4^ cells/mL) cells were cultured for 24 h, followed by treatment with increasing doses of TMZ (0–5000 µM) or CDDP (0–5 µM). After 48 h, the cells were incubated with the cell counting kit-8 reagent, which was diluted 1:10 into the culture medium, and the absorbance of the medium was measured at 450 and 650 nm after 4 h of incubation. Data are presented as means ± standard deviation (*n* = 3). Inhibitory concentration 50 (IC50).

First, we determined the half maximal inhibitory concentrations of TMZ and CDDP to compare the sensitivity of glioblastoma cells with TMZ, a standard treatment option for glioblastoma, and CDDP that is not currently used for glioblastoma treatment. As shown in Figure 1, the half maximal inhibitory concentration was 1.5 µM for CDDP and 2144 µM for TMZ (Figure 1), indicating that the cytotoxic effect of CDDP in glioblastoma cells was higher than that of TMZ and supporting our hypothesis that enhanced BBB permeability of CDDP could improve its therapeutic efficacy in glioblastoma.

### 2.2. CDDP Delivery to Glioblastoma

**Figure 2:**
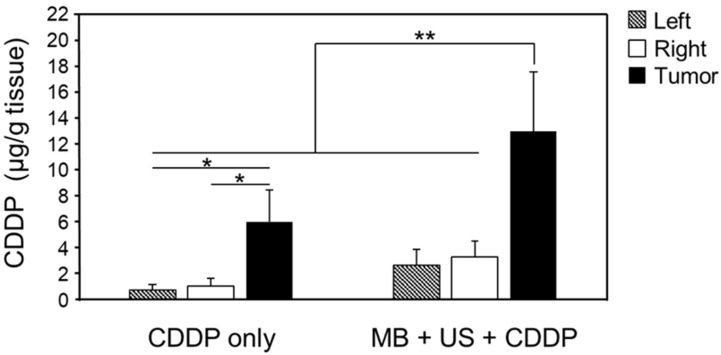
Amount of CDDP delivered to various parts of the brain. Twelve days after tumor inoculation, mice were treated with CDDP combined with MBs and US or with CDDP alone. One hour after treatment, the mice were perfused with phosphate-buffered saline. Brains were collected. The left hemisphere (Left), right hemisphere not containing the tumor (Right), and tumor (Tumor) specimens were prepared. CDDP concentrations were measured using inductively coupled plasma mass spectrometry. Data are presented as means ± standard deviation (*n* = 5, Tukey–Kramer test, * *p* < 0.05; ** *p* < 0.01).

Based on our initial findings suggesting that CDDP could be used for glioblastoma therapy, we next evaluated the delivery of CDDP to the brain by opening the BBB using MBs with US. To this end, mouse glioblastoma cells were transplanted into the brains of mice to create a glioblastoma model. The mice were then intravenously administered a mixture of CDDP and MBs, which was immediately followed by US or 1 h after administration of CDDP only. CDDP concentrations were measured in both groups. As shown in Figure 2, the CDDP concentration at the tumor site was significantly higher in mice administered CDDP together with MBs and US than in those administered CDDP alone. The higher CDDP concentration at the tumor site compared with the other two sites in the same mice in the group of animals that were administered CDDP alone was considered to be due to the heterogeneous tumor vessel structure. In mice administered CDDP together with MBs and US, the higher CDDP concentration at the tumor site compared with the other two sites in the same mice suggested that CDDP could be selectively delivered to the target site, i.e., the tumor. When the combination of CDDP, MBs and US was applied, the CDDP concentration in the left and right side of the brain tended to increase compared with that of CDDP alone (Figure 2). We considered that the reflection of US in the skull might be induced because the skull of mouse is small, and the permeability of BBB would be increased. It is known that CDDP induces leukoencephalopathy by the damage in the cerebral white matter as a potential side effect. Since symptoms of leukoencephalopathy are mainly observed the abnormal behavior such as gait disturbance, we are planning behavioral experiments after the treatment in future studies. On the other hand, the effect of US reflection in the human brain would be reduced due to the larger size of the brain. In any case, we need to evaluate the side effects derived from increased CDDP in the normal brain.

Although we did not examine CDDP concentration after the treatment of US alone, we have previously reported that the treatment of US alone did not enhance the permeability of BBB [26]. In this study, we used same condition of US exposure with previous report, and we considered that the effect of US alone on BBB might be limited.

**Figure 3:**
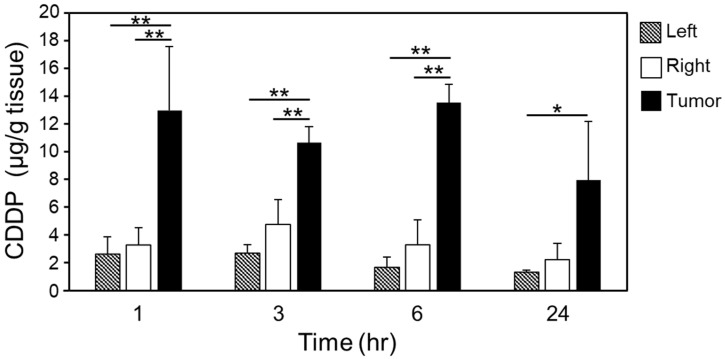
CDDP delivery over time following treatment. Brains were collected 1, 3, 6, and 24 h after treatment and perfused with phosphate-buffered saline. The left hemisphere (Left), right hemisphere not containing the tumor (Right), and tumor (Tumor) specimens were prepared. CDDP concentrations were measured using inductively coupled plasma mass spectrometry. Data are presented as means ± standard deviation (*n* = 3, Tukey–Kramer test, * *p* < 0.05; ** *p* < 0.01).

It is possible that the residual time in the brain after CDDP administration could impact its therapeutic effect. Therefore, time-dependent changes in CDDP concentration in the three brain sites were determined by measuring CDDP levels 1, 3, 6, and 24 h after its administration in combination with MBs and US. Although the concentration of CDDP delivered to the tumor site remained consistent after 24 h (Figure 3), the plasma CDDP concentration decreased rapidly immediately after administration. Additionally, at 1 h after treatment, the CDDP concentration at the tumor site was higher in the CDDP group treated with MBs and US than in the CDDP-only group. Furthermore, the CDDP concentration at the tumor site remained high in the CDDP group treated with MBs and US even 24 h after treatment. The presence of multidrug resistance-associated protein (MRP) in the BBB and its involvement in the excretion of CDDP inhibits the delivery of CDDP to the brain [29,30]. In the present study, opening the BBB using MBs in combination with US facilitated the delivery of CDDP beyond the vascular endothelial cells and into the glioblastoma, which allowed CDDP to remain on site for an extended time period without its excretion by MRP.

The comparison of CDDP concentrations between the tumor tissue and plasma revealed that CDDP quickly disappeared in plasma within 1 h of treatment. In contrast, CDDP was present in the tumor tissue 24 h after its administration, indicating that CDDP, once delivered to the glioblastoma tissue, was not easily released into the peripheral blood. Besides MRP, other transporters, such as p-glycoprotein (p-gp) and breast cancer resistance protein (BCRP), are also expressed at the BBB [31]. The BBB-opening method using MBs combined with US could be considered to prolong the availability of other anticancer drugs that are substrates for those transporters at the glioblastoma site.

### 2.3. Recovery of BBB Opening

**Figure 4:**
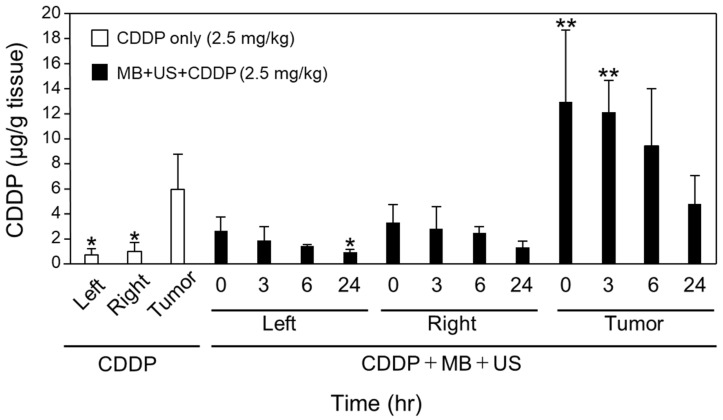
BBB opening time in a tumor mouse model. MBs were administered into the tail vein and immediately irradiated with US 12 days after tumor inoculation. CDDP was administered into the tail vein 0, 3, 6, and 24 h after MB and US treatment. The mice were perfused with phosphate-buffered saline 1 h after treatment, and brains were collected. The left hemisphere (Left), right hemisphere not containing the tumor (Right), and tumor (Tumor) specimens were prepared. CDDP concentrations were measured using inductively coupled plasma mass spectrometry, and the CDDP levels at each time point were compared with those in the CDDP only group (Tomor). Data are presented as means ± standard deviation (*n* = 3, Dunnett’s test (vs. CDDP only (Tumor)), * *p* < 0.05; ** *p* < 0.01).

The combination of MBs and US enhanced BBB permeability and resulted in effective CDDP delivery. This enhancement may be caused by opening the tight junction between endothelial cells in the brain by mechanical movements, such as oscillation of MBs under US irradiation. Zhao et al. reported that the tight junction was opened, and the gap between the cells became wide, as observed under an electron microscope [32]. Although the BBB-opening technique is an effective strategy for drug delivery to the brain, there may be a risk of allowing undesigned molecules and pathogens to access the brain. Therefore, the recovery of the tight junction after BBB opening by MBs and US irradiation was assessed (Figure 4). In this experiment, CDDP delivery to the brain by CDDP injection was examined each time after MBs and US irradiation. CDDP predominantly increased only at 0 h and 3 h in the CDDP group treated with MBs and US compared with that in the CDDP-only group. Thus, BBB permeability increased in the tumor tissue area by the combination of MBs and US for at least 3 h and returned to the initial level after 6 h (Figure 4). These results suggest that BBB opening using the combination of MBs and US was a transient event. In the brain, claudin-5 makes a tight junction between endothelial cells and functions as a BBB [33]. The half-life of claudin-5 is approximately 90 min [34]. Claudin-5 should be renewed to maintain its function as a BBB. Therefore, the tight junction disrupted by the mechanical effect of MBs under US irradiation is believed to be repaired quickly.

A small amount of CDDP was observed in brain areas other than those directly irradiated by US. This finding suggests that the BBB-opening effect was very low in the area without direct US irradiation. Therefore, the leakage of large molecules, such as proteins in blood flow into the brain, was believed to be limited to normal brain tissue. Even in the tumor tissue area, BBB opening was a transient event. Our previous study reported no direct brain damage or hemorrhage after treatment with MBs and US. Overall, we concluded that our BBB-opening system by combining MBs with US would be a safe system for drug delivery to the brain.

### 2.4. Blood Urea Nitrogen (BUN), Aspartate Aminotransferase (AST), and Alanine Aminotransferase (ALT) Evaluation following CDDP Administration

**Figure 5:**
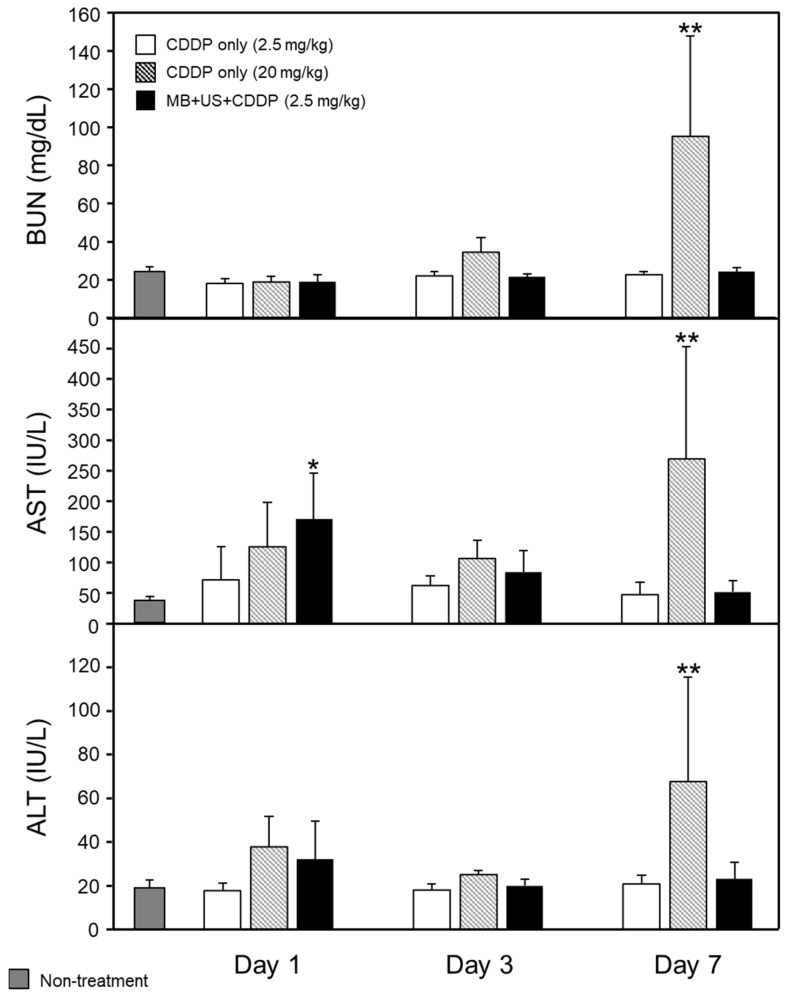
Evaluation of renal and hepatic function with CDDP. Normal mice were administered MBs (3 × 10^9^/kg) and CDDP (20 mg/kg or 2.5 mg/kg) intravenously into the tail vein and immediately irradiated with US (frequency 3 MHz, irradiation intensity 0.5 W/cm^2^, duty cycle 50%, pulse repetition frequency 10 Hz, and irradiation time 3 min). Blood was collected 1, 3, and 7 days later. The blood was then allowed to clot for 60 min and centrifuged at 1700× *g* for 15 min at 24 °C. Serum was collected for (A) BUN, (B) AST, and (C) ALT measurements. Data are presented as means ± standard deviation (*n* = 4, Dunnett’s test (vs. Non-treatment), * *p* < 0.05; ** *p* < 0.01).

The serum levels of BUN, AST, and ALT, which are known indicators of renal and hepatic function that may be affected by CDDP [17], were measured to examine the adverse effects of CDDP (Figure 5). The BUN, AST, and ALT levels significantly increased 7 days after the injection of CDDP (20 mg/kg) as the positive control. This finding suggests that an excess CDDP dose induces severe renal and hepatic toxicity. In mice injected with CDDP (2.5 mg/kg) only, BUN, AST, and ALT levels did not increase. AST levels temporally increased 1 day after CDDP injection (2.5 mg/kg) combined with MBs and US. However, the AST levels decreased to the level of the untreated mice 3 and 7 days after CDDP injection (2.5 mg/kg) combined with MBs and US. Although a slight increase in the AST level was observed in the mice with CDDP injection (2.5 mg/kg), this increase was transient, and the ALT level was not significant. These findings suggest that the hepatic toxicity in this treatment is not severe, and CDDP delivery to the brain by BBB opening gains an advantage over the slight adverse effects of transient hepatic toxicity. Thus, CDDP treatment with this BBB-opening technology is expected not to induce severe toxicity in the kidney and liver.

### 2.5. Therapeutic Effect of CDDP in a Mouse Model of Glioblastoma

**Figure 6:**
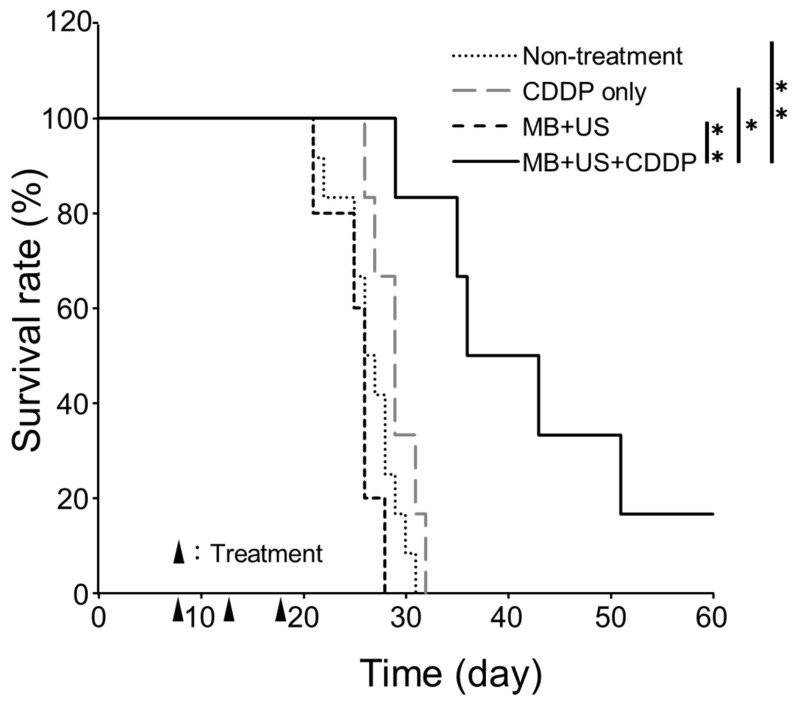
Comparison of survival in specific treatment groups in a mouse model of glioblastoma. The mice were treated with cisplatin delivered with MBs and ultrasound (MB + US + CDDP), MBs and US alone (MB + US), cisplatin alone (CDDP), or left untreated (Non-treatment) 7 days after tumor transplantation. Data are presented as means ± standard deviation (*n* = 6, logrank test, * *p* < 0.05; ** *p* < 0.01).

The delivery of CDDP to the glioblastoma tissue is expected to have a beneficial therapeutic effect. As shown in Figure 6, the survival was significantly longer in mice with glioblastoma treated with CDDP delivered with MBs and US than in untreated mice with glioblastoma, those with glioblastoma treated with CDDP alone, and those with glioblastoma treated with MBs and US alone. This finding indicated that the combination of MBs and US enhanced the antitumor effect of CDDP by prolonging its availability at the glioblastoma site. Drug delivery to the brain is important for the treatment of various brain diseases, including glioblastoma. In the present study, we tested the efficacy of our novel BBB-opening method in glioblastoma, which has a low clinical response rate and a high mortality rate [6,7]. 

We focused on CDDP, a versatile drug used to treat more than 50% of all cancers. Our analysis revealed that glioblastoma was more sensitive to CDDP than to TMZ, suggesting that CDDP may be an effective therapeutic approach in glioblastoma. Given the limited access of CDDP to the brain, we utilized a noninvasive and efficient drug delivery method, which was based on the optimization of the lipid composition and inner gas of MB and the US irradiation conditions [26,27]. In experiments utilizing this method to deliver CDDP to mice with glioblastoma, we observed that the CDDP concentrations were highest in the group that were treated with CDDP that was delivered using MBs in combination with US. Although the CDDP concentrations were also enhanced in the tumor tissue of mice that were treated with CDDP without the BBB-opening method, the survival benefit was observed only in the group of mice treated with CDDP using MBs in combination with US. This finding indicated that the amount of CDDP delivered to the tumor tissue was not sufficient to exert a therapeutic effect in the mice that were treated without the MBs and US and that the combination of MBs and US was necessary. In addition, the BBB-opening method using MBs and US might cause injury to brain tissue depending on the US irradiation conditions. However, in this study, no increase in survival was observed in mice treated with MBs and US, suggesting that MBs and US were unlikely to damage the tumor tissue. These results suggested that the prolonged survival was due to the antitumor effect of CDDP delivered to the glioblastoma by opening the BBB using MBs and US in the absence of off-target tissue damage. In addition, the amount of CDDP delivered to the tumor tissue was higher in the mice treated with CDDP delivered using MBs and US than in those treated with the same dose of CDDP without opening the BBB. CDDP is an effective anticancer drug used to treat many solid tumors. However, CDDP is associated with systemic side effects, such as dose-limiting toxicity, nephrotoxicity, neurotoxicity, and myelosuppression [35,36] and excessive CDDP administration may have serious side effects. The study results indicated that MBs and US could prevent CDDP overdose and reduce its systemic side effects.

Drug resistance is a common problem in cancer therapy with anticancer drugs [35]. In the brain, transporters, such as p-gp, BCRP, and MRP, express and function as exhaust pumps to prevent drugs from entering the brain [31]. Our delivery system with MBs and US is expected to be applied to anticancer drugs that are excreted by these transporters and have low permeability to the brain, as well as CDDP. Therefore, using a variety of anticancer drugs could help overcome the problem of drug resistance to anticancer drugs in each patient. Generally, tumor tissue has heterogeneous vascular structures that make antitumor drug delivery difficult. Our delivery system is based on the mechanical effect of MBs under US irradiation. Therefore, the combination of MBs and US is expected to be easily applied to various tumor tissue types. However, further studies are needed to develop approaches to precisely control the position of US irradiation, thereby improving the efficacy of drug delivery to the brain and the efficacy of treatment.

## 3. Materials and Methods

### 3.1. Reagents

CDDP was purchased from Nichi-Iko Pharmaceutical, Toyama, Japan. TMZ was purchased from Merck, Tokyo, Japan; 1,2-distearoyl-sn-glycero-3-phosphocholine (DSPC), 1,2-distearoyl-sn-glycero-3-phosphoglycerol (DSPG) and N-(carbonyl-methoxypolyethyleneglycol-2000)-1,2-distearoyl-sn-glycero-3-phosphoethanolamine (DSPE-PEG2000) were purchased from NOF Corporation, Tokyo, Japan. Perfluoropropane (C_3_F_8_) was purchased from Takachiho Chemical, Tokyo, Japan. Cell counting kit-8 was purchased from Dojindo, Kumamoto, Japan. Other reagents were obtained from FUJIFILM Wako Pure Chemical, Osaka, Japan.

### 3.2. Cell Culture

A lentiviral vector carrying the green fluorescent protein (GFP)-luciferase (Luc) gene fragment and the puromycin resistance gene was transfected into glioma-261 cells, a model of glioblastoma, and the transfected cells were cultured in a medium supplemented with puromycin. The cells were screened for the presence of GFP, puromycin resistance gene, and luciferase, and those harboring all three were cultured in Roswell Park Memorial Institute 1640 medium containing 10% fetal bovine serum and puromycin (0.5 μg/mL) at 37 °C with 5% CO_2_.

### 3.3. Animals

Six-week-old female C57BL/6J mice and six-week-old male ddY mice were purchased from Sankyo Labo Service Corporation (Tokyo, Japan). All animal experiments were performed in accordance with the Ethical Code for Animal Experiments of Teikyo University and approved by the Institutional Animal Care and Use Committee of Teikyo University.

### 3.4. Evaluation of Viability

Glioma-261 GFP-Luc (3 × 10^4^ cells/mL) cells were cultured at 37 °C for 24 h. Next, the medium was removed, and the cells were incubated in a medium supplemented with TMZ (0–5000 µmol/L) or CDDP (0–5 µmol/L). After 48 h, the medium was removed, and the cells were incubated with the cell counting kit-8, which was diluted 1:10 into the culture medium. After incubation for 4 h, the absorbance of the medium was measured at 450 and 650 nm.

### 3.5. Preparation of MBs

Liposomes composed of DSPC, DSPG, and DSPE-PEG2000 at a molar ratio of 30:60:10 were prepared using the lipid film hydration method [27]. Briefly, a lipid film was prepared using a rotary evaporator, followed by drying overnight in a vacuum desiccator to completely remove the solvents. The lipid film was hydrated with 100 mM NaH_2_PO_4_ (pH 7.4, 30 mL) to a lipid concentration of 10 mM. After hydration, the liposome suspension was prepared by shaking (65 °C, 30 min, 160 rpm) in a constant-temperature shaking bath (NTS-4000; WAKENYAKU CO., LTD., Japan). The liposome suspension was then sonicated (42 kHz) for 10 min in a bath-type sonicator (2510J-DTH; Yamato Scientific Co., Ltd., Tokyo, Japan); 100 mM NaH_2_PO_4_ (pH 7.4, 270 mL) was added to bring the lipid concentration in the liposome suspension to 1 mM and placed in a homogenizer (Labolution Mark II 2.5; PRIMIX Corporation, Hyogo, Japan) vessel. The vessel was replaced with C_3_F_8_ and sealed. The liposome suspension and C_3_F_8_ in the vessel were then stirred with a homogenizer (40 °C, 60 min, 7500 rpm) to prepare the MB suspension. The MB dispersion was mixed with 18% sucrose solution at a 1:1 (*v*/*v*) ratio, and 2 mL of the mixture was dispensed into a 5 mL vial. The air in the headspace was replaced with C_3_F_8_, and the vial was closed with a rubber lid, followed by freezing at −30 °C. Next, the rubber lid was opened, and freeze-drying was performed at −30 °C for 1 h, −20 °C for 72 h, and 20 °C for 48 h using a freeze dryer with a shelf temperature-controlled drying chamber (EYELA FDU-1100 and EYELA DRC-1100; Tokyo Rikakikai, Tokyo, Japan). The vial was closed with a rubber lid under vacuum and capped with an aluminum cap. Before use in experiments, the freeze-dried MBs were reconstituted in 2 mL of ultrapure water and briefly shaken. The resultant gas-loaded lipid-based MBs were used in experiments. The average diameter of the MBs was approximately 1.4 µm based on number distribution. We have optimized the lipid outer shell and inner gas of the MBs in terms of their stability and drug delivery efficiency to the brain [27,28]. In this study, the optimized MBs were utilized.

### 3.6. CDDP Delivery to Glioblastoma

For tumor inoculation, mice were anesthetized with an anesthetic mix containing 0.3 mg/kg medetomidine hydrochloride (Domitol^®^; 1 mg/mL), 4 mg/kg midazolam (5 mg/mL), and 5 mg/kg butorphanol tartrate (Betrufal^®^; 5 mg/mL). Next, the head of the mouse was fixed to a stereotaxic apparatus (SR-6M-HT; NARISHIGEP). An incision was made on the skin to expose the skull, and a hole was made 2 mm to the right and 1 mm in front of the bregma. A total of 2 × 10^5^ glioma-261 GFP-Luc cells in 5 µL PBS were transplanted at a depth of 3 mm through the hole. Twelve days after tumor inoculation, a mixture of MBs (3 × 10^9^ particles/kg) and CDDP (2.5 mg/kg) was administered into the tail vein of the mouse; immediately, the mouse was irradiated at the injection site with US (frequency, 3 MHz; irradiation intensity, 0.5 W/cm^2^; duty cycle, 50%; pulse repetition frequency, 50 Hz; irradiation duration, 3 min). Another group of animals were administered CDDP 12 days after the tumor inoculation. In both groups, 1, 3, 6, and 24 h after each treatment, the mice were sacrificed with blood perfusion, and the brain was removed under deep anesthesia. The isolated whole brains were divided into three specimens: one specimen containing the tumor in the right hemisphere, one specimen containing the right hemisphere without the tumor, and one specimen containing the left hemisphere. Then, 50 mg brain tissue was removed from each specimen and placed in tetrafluoromethoxylene inserts with indium (350 ng/mL), which were used as internal standards, and 70% nitric acid. The inserts were placed in an ETHOS D pretreatment unit (Milestone General, Kanagawa, Japan) to ash the samples, which were then diluted 35-fold with ultrapure water. Inductively coupled plasma mass spectrometry (iCAP Q; Thermo Fisher Scientific, Tokyo, Japan) was used to detect the trace amounts of platinum in the CDDP to calculate CDDP content.

### 3.7. Evaluation of Renal and Hepatic Function after CDDP Administration

The adverse effects of mice administered a therapeutic CDDP dose (2.5 mg/kg) or an excess dose of CDDP (20 mg/kg) as a positive control were assessed. After shaving the hair on the head of normal 6-week-old male ddY mice, they were intravenously administered MBs (3 × 10^9^/kg) and CDDP (2.5 mg/kg) into the tail vein and immediately irradiated with US (3 MHz frequency, 0.5 W/cm^2^ intensity, 50% duty cycle, 10 Hz pulse repetition frequency, and 3 min duration). A group of mice administered CDDP (2.5 or 20 mg/kg) alone was also prepared. After 1, 3, and 7 days, blood was collected from mice using cardiac puncture. The blood was then allowed to clot for 60 min and centrifuged at 1700× *g* for 15 min at 24 °C, and serum was collected. Serum levels of BUN, AST, and ALT were measured using Oriental Yeast Co., ltd. (Shiga, Japan).

### 3.8. Evaluation of Survival following CDDP Delivery to the Glioblastoma Using MBs and US

Seven days after tumor inoculation, mice were injected with a mixture of MBs (3 × 10^9^ particles/kg) and CDDP (2.5 mg/kg) and immediately irradiated at the cell injection site with US. The treatment was repeated thrice every 5 days to record the survival of animals.

## 4. Conclusions

In conclusion, the combination of MBs and US successfully delivered CDDP, whose physiochemical properties prevent its delivery to the brain across the BBB. Furthermore, CDDP delivered through this method exhibited an antitumor effect in a mouse model of glioblastoma. Therefore, this study showed that CDDP, if delivered effectively, would be an innovative treatment option for glioblastoma and expand the diversity of glioblastoma treatment. As an innovative drug delivery system, the BBB-opening method evaluated in the present study should be evaluated as a new option in the treatment of patients with glioblastoma as well as in other glioblastoma and central nervous system disorders in future studies.

## Data Availability

Data is contained within the article.
